# Supplementary figures and images for: Alpha- and beta-adrenergic octopamine receptors in muscle and heart are required for Drosophila exercise adaptations
Source: PLoS Genet. 2020 Jun 24;16(6):e1008778. doi: 10.1371/journal.pgen.1008778 (PMC7351206; doi:10.1371/journal.pgen.1008778)

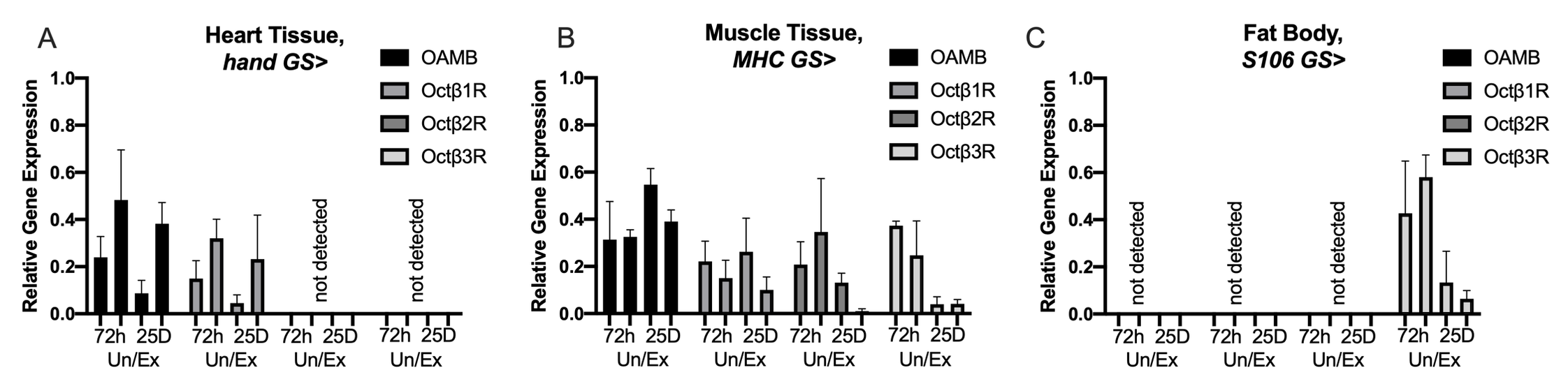

Supplement: S1 Fig — Bars represent triplicate samples consisting of (A) 20 hearts, or samples from 5 flies consisting of (B) IFM or (C) adult fat body. Samples were assigned to exercised and unexercised groups and collected at 72 hours, prior to the first endurance test, and 25 days, after the conclusion of exercise training. qRT-PCR was performed after cDNA isolation from aforementioned tissues. Relative expression is calculated as ΔΔCT and analyzed using ANOVA with Tukey post-hoc. Gene expression is expressed in relation to uninduced RU- controls. See methods for primer sequences, isolation, purification and reaction conditions. (TIFF) [file pgen.1008778.s001.tiff]

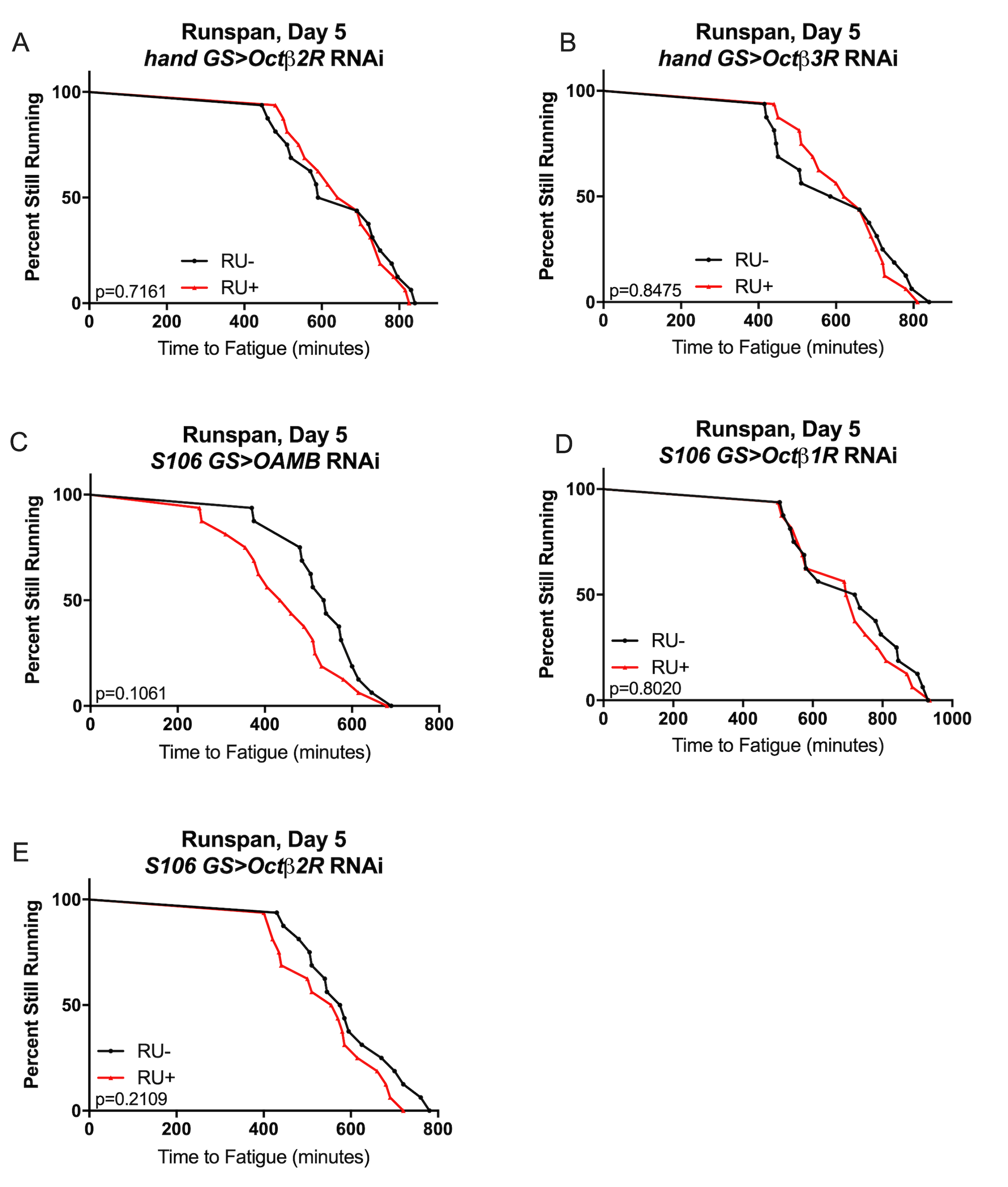

Supplement: S2 Fig — Neither Octβ2R nor Octβ3R were detected in adult Drosophila heart (A, B), and OAMB, Octβ1R and Octβ2R (C-E) transcripts were not detectable in adult fat body. Day 5 endurance in RU+ flies of each of the aforementioned genotypes were not statistically different from their RU- control flies of the same age. (TIFF) [file pgen.1008778.s002.tiff]

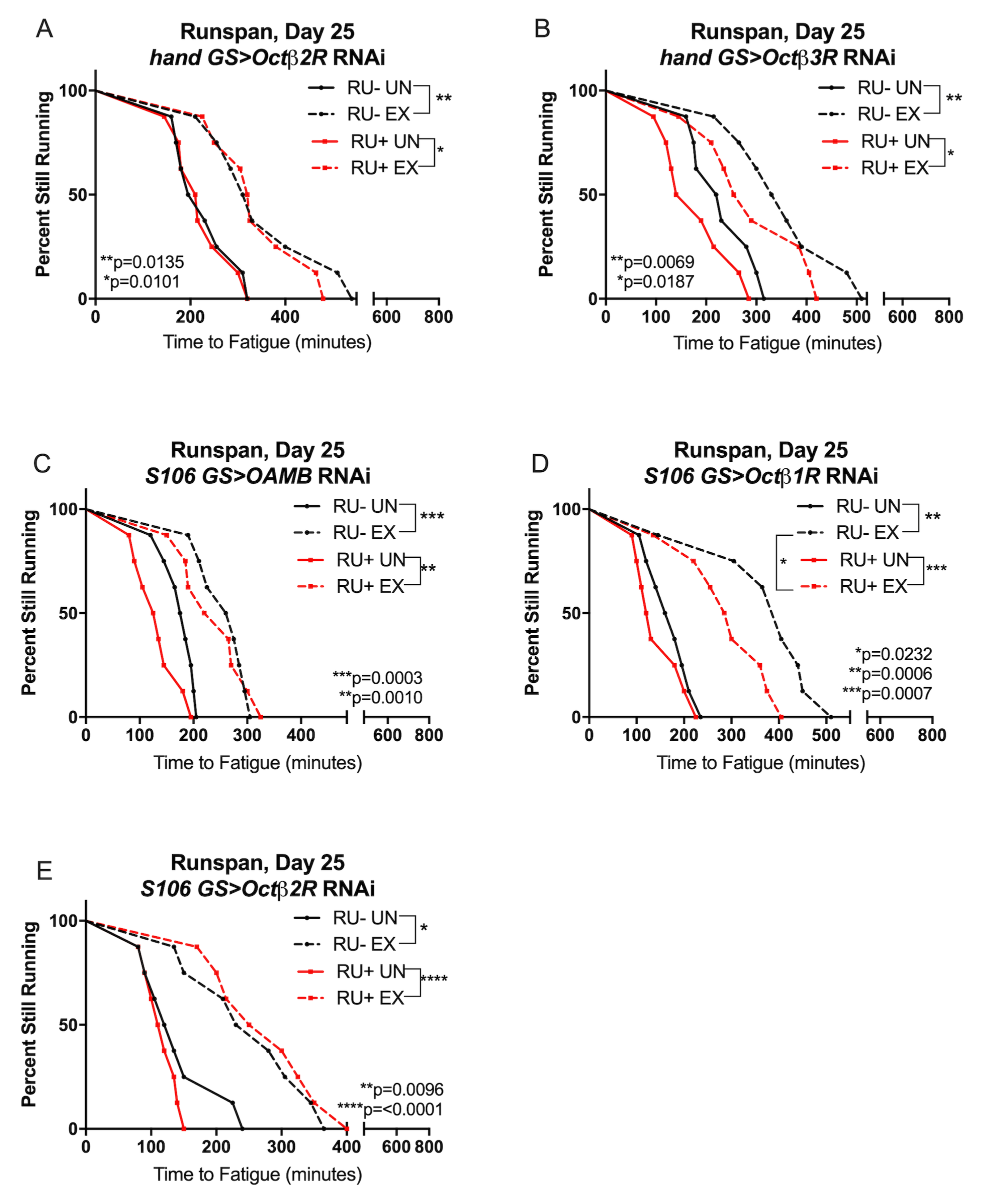

Supplement: S3 Fig — hand GS>Octβ2R RNAi and hand GS>Octβ3R RNAi RU+ flies respond to exercise with improved endurance (A, B) as do S106 GS>OAMB RNAi, S106 GS>Octβ1R RNAi and S106 GS>Octβ2R RNAi flies (C-E). (log-rank, p-values indicated in panels). (TIFF) [file pgen.1008778.s003.tiff]

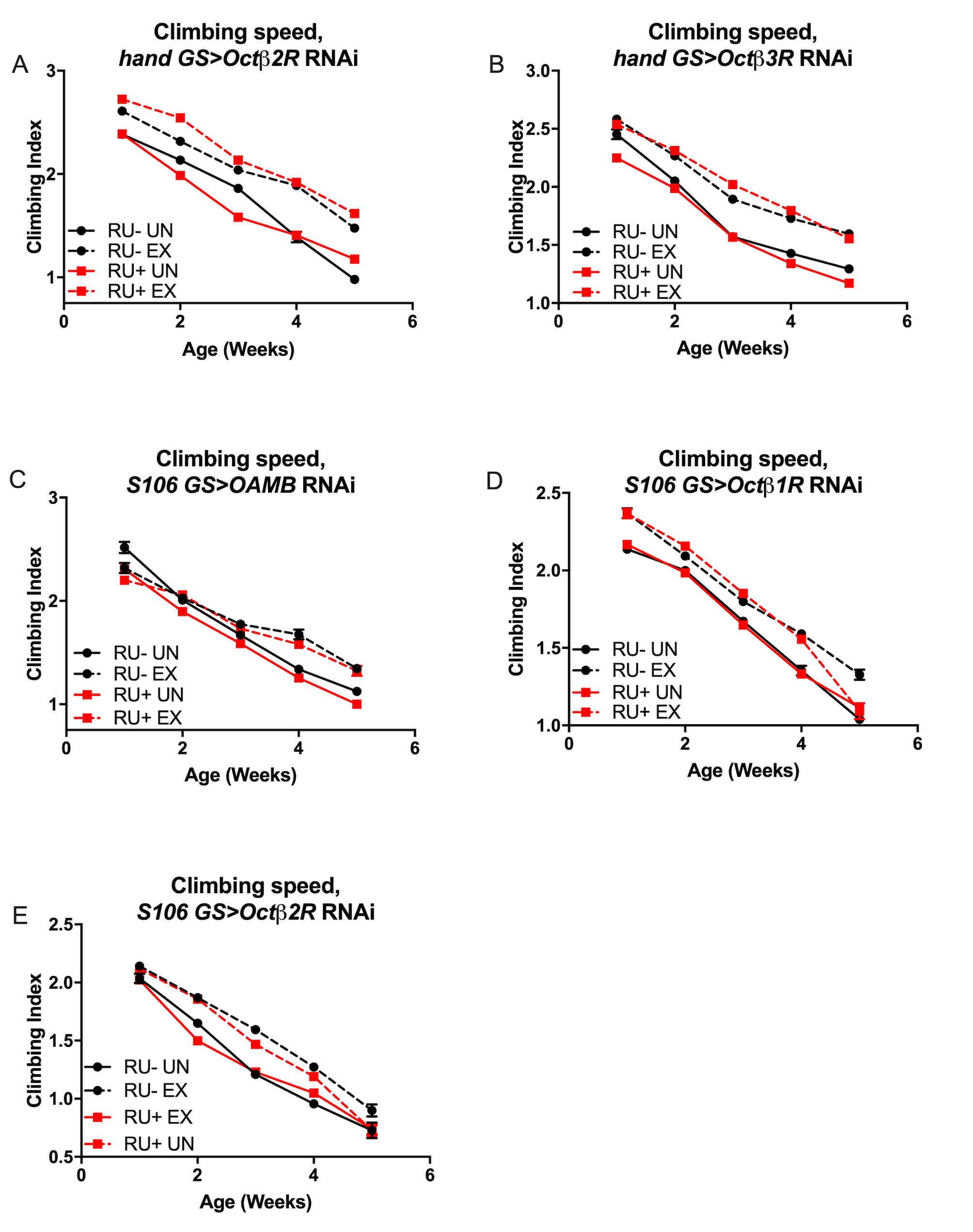

Supplement: S4 Fig — Both RU+ flies and uninduced RU- controls respond to exercise training with faster climbing speed across ages in (A) hand GS>Octβ2R RNAi, (B) hand GS>Octβ3R RNAi (C) S106 GS>OAMB RNAi, (D) S106 GS>Octβ1R RNAi and (E) S106 GS>Octβ2R RNAi groups. (2-way ANOVA, exercise effect, p<0.0001 after week 2, all groups). (TIFF) [file pgen.1008778.s004.tiff]

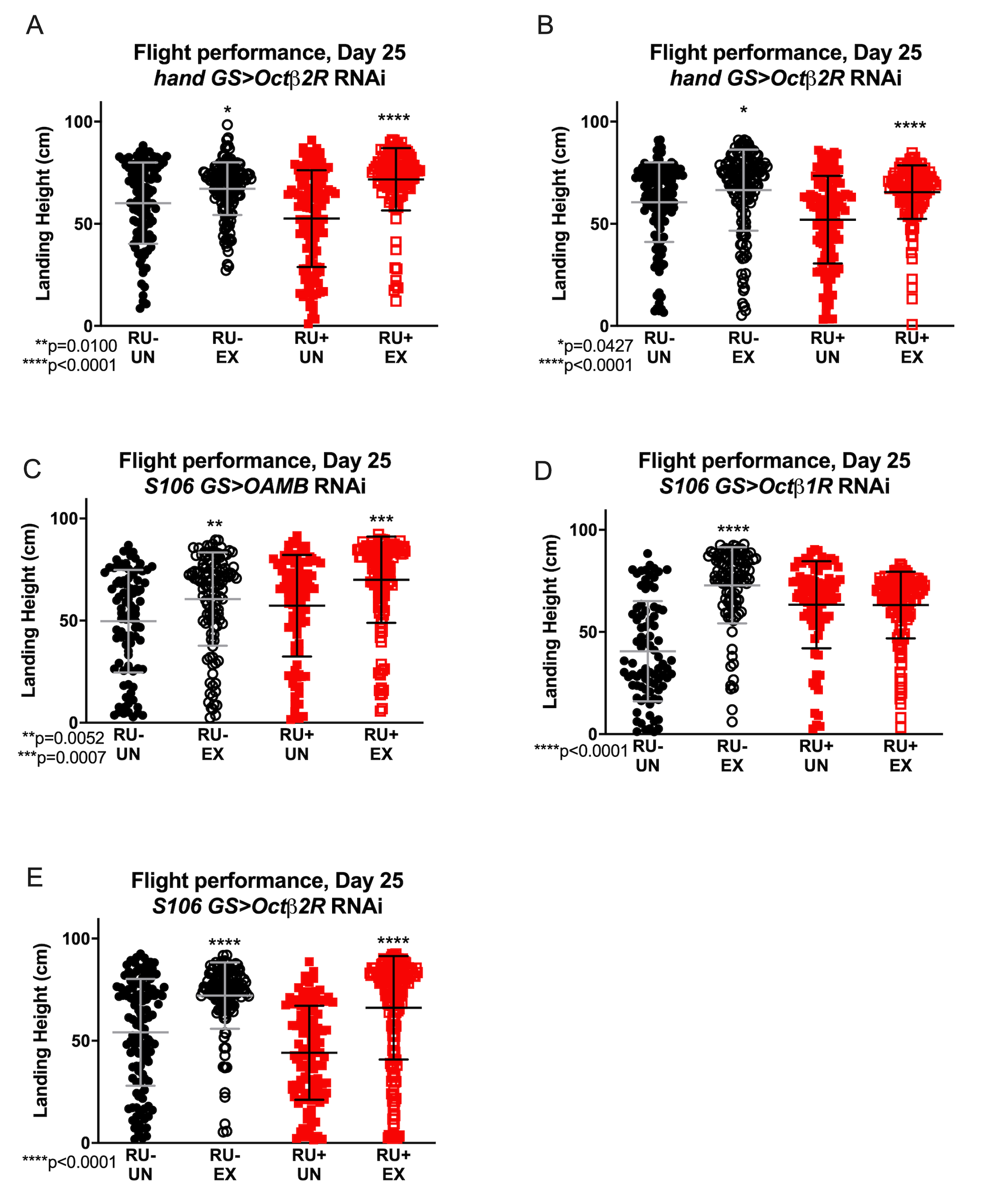

Supplement: S5 Fig — Landing height is higher in exercise trained RU- and RU+ A) hand GS>Octβ2R RNAi, (B) hand GS>Octβ3R RNAi (C) S106 GS>OAMB RNAi, (D) S106 GS>Octβ1R RNAi and (E) S106 GS>Octβ2R RNAi flies. (ANOVA with Tukey multiple comparisons, p values indicated in panels). (TIFF) [file pgen.1008778.s005.tiff]

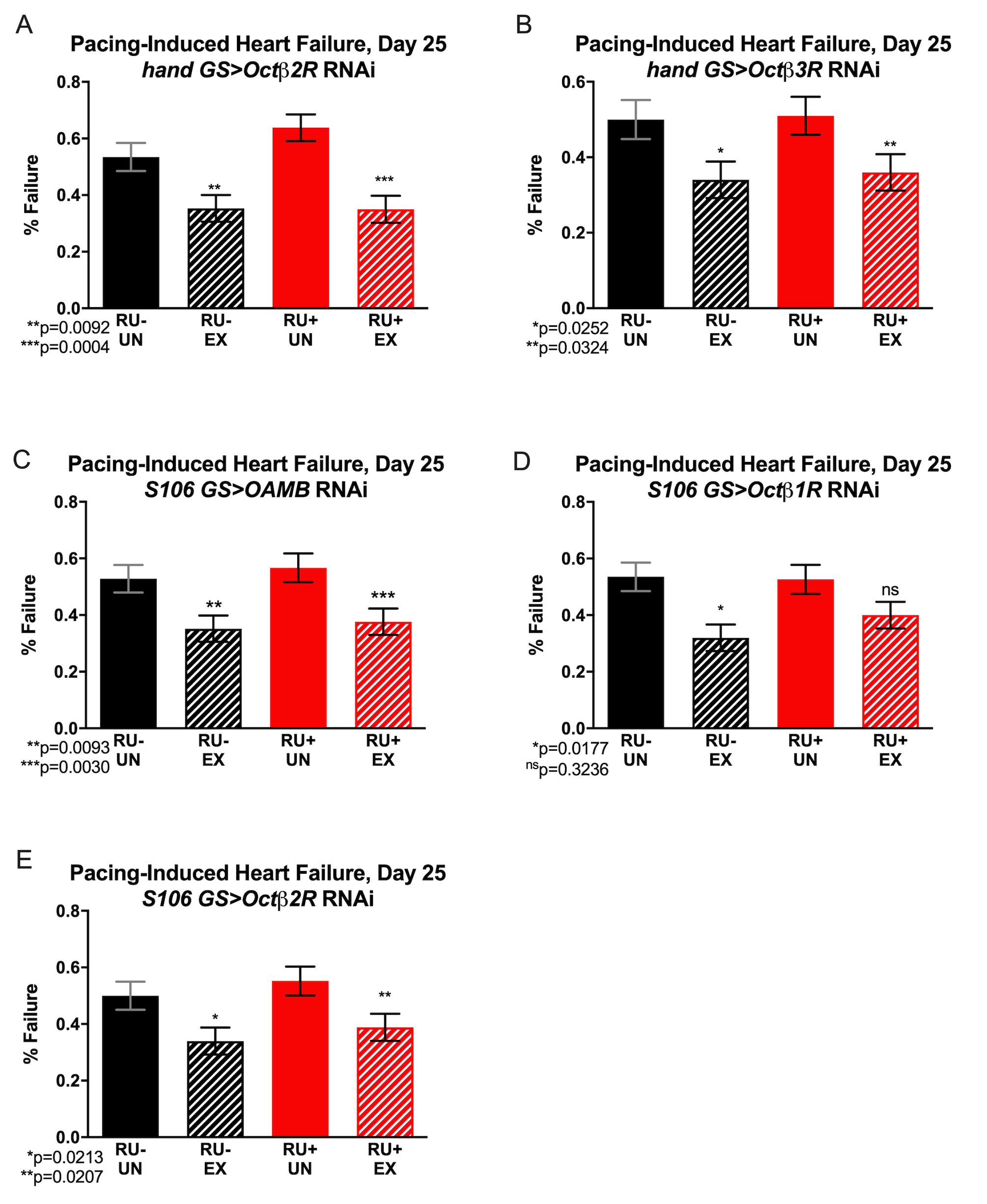

Supplement: S6 Fig — hand GS>Octβ2R RNAi and hand GS>Octβ3R RNAi RU+ flies respond to exercise with improved tolerance to external cardiac pacing (A, B) as do S106 GS>OAMB RNAi, S106 GS>Octβ1R RNAi and S106 GS>Octβ2R RNAi flies (C-E). (Chi-squared, p values indicated in panels). (TIFF) [file pgen.1008778.s006.tiff]

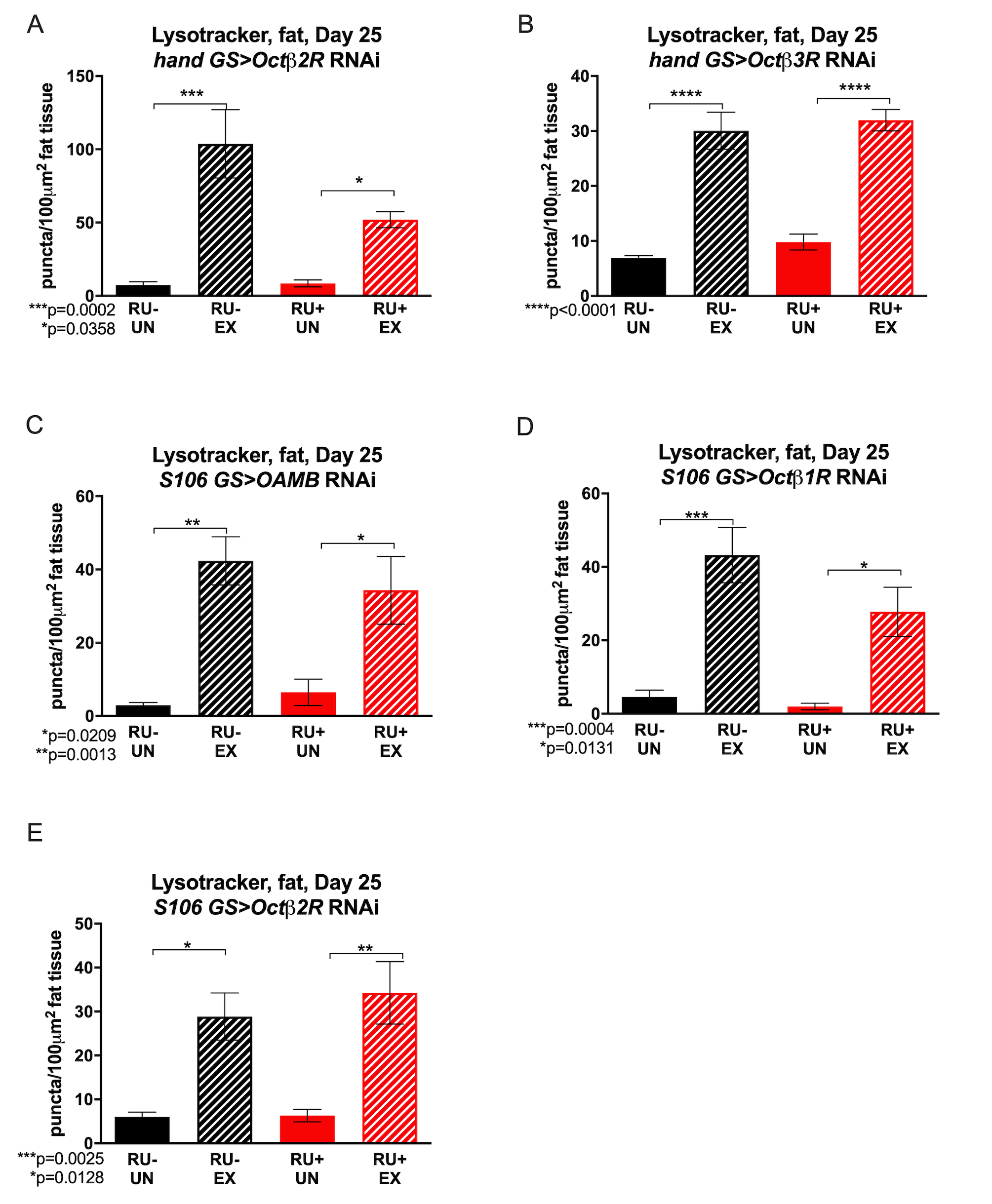

Supplement: S7 Fig — LysoTracker staining is higher in exercise trained RU- and RU+ A) hand GS>Octβ2R RNAi, (B) hand GS>Octβ3R RNAi (C) S106 GS>OAMB RNAi, (D) S106 GS>Octβ1R RNAi and (E) S106 GS>Octβ2R RNAi flies. (ANOVA with Tukey multiple comparisons, p values indicated in panels). (TIFF) [file pgen.1008778.s007.tiff]

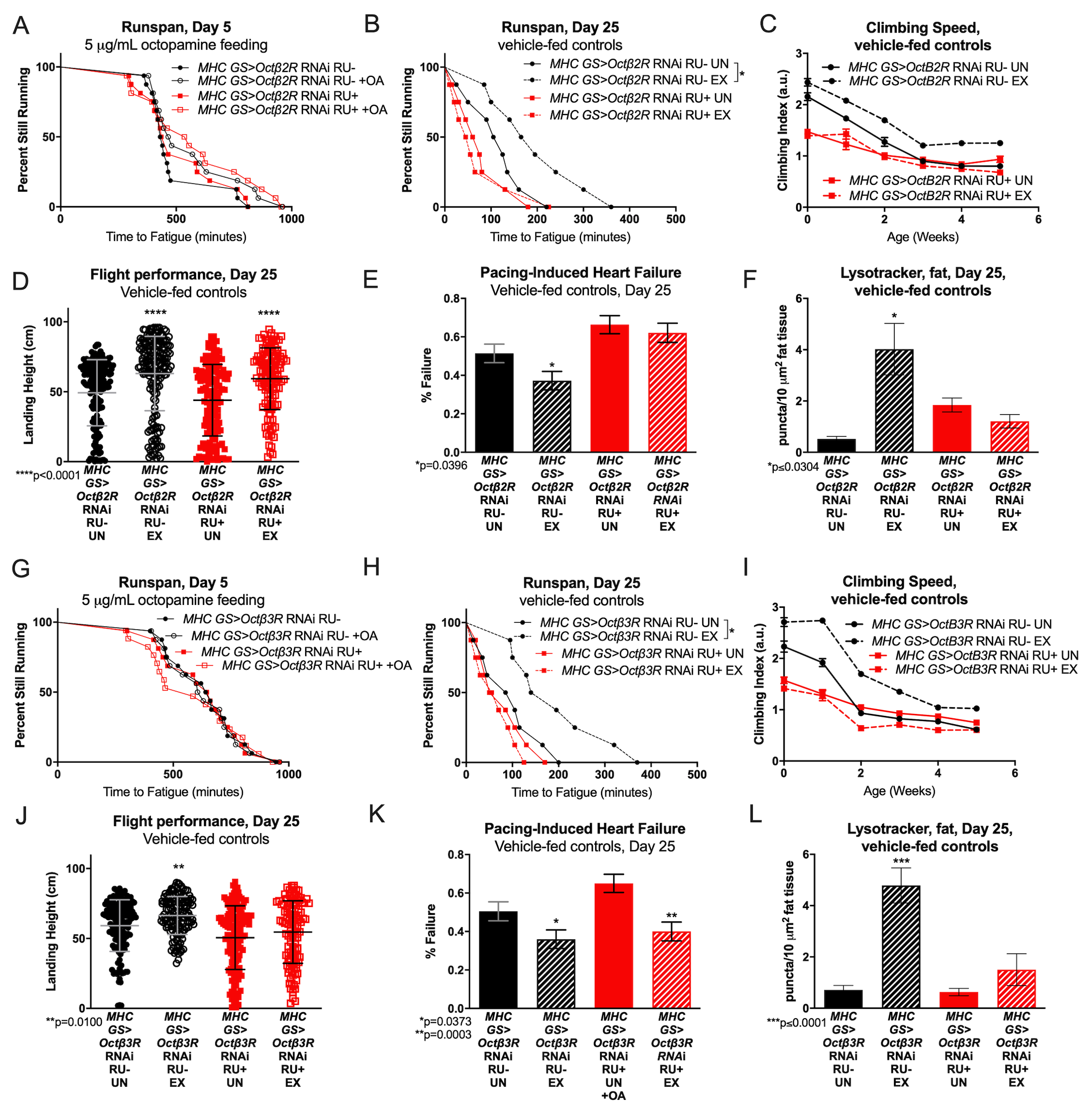

Supplement: S8 Fig — (A) MHC GS>Octβ2R RNAi RU- and RU+ flies fed 5μM OA or vehicle for 72 hours have equivalent endurance at day 5-post eclosion (log-rank, p = 0.2790, n = 16 vials of 20 flies for each cohort). (B) OA-fed MHC GS>Octβ2R RNAi RU+ flies have endurance similar to untrained, vehicle-fed RU- flies whether exercised or not (log-rank, p≥0.2558). Uninduced, vehicle-fed exercised controls retain better endurance than unexercised siblings (log-rank, p = 0.0439, n = 8 vials of 20 flies for all cohorts). (C) Exercise-trained, vehicle-fed MHC GS>Octβ2R RNAi RU- flies have faster climbing than unexercised, vehicle-fed siblings across ages (2-way ANOVA, exercise effect, p<0.0001). Both exercised and unexercised vehicle-fed MHC GS>Octβ2R RNAi have reduced climbing speed in comparison to RU- groups up to the second week of training (2-way ANOVA, genotype effect, p<0.0001) and do not improve with training or vehicle feeding, having similar climbing speed to untrained, RU- vehicle-fed groups in later weeks (n≥100 for all cohorts, error bars = SEM). (D) Vehicle feeding does not affect adaptation to flight performance after exercise in either MHC GS>Octβ2R RNAi RU- or MHC GS>Octβ2R RNAi RU+ flies, as both increase landing height in comparison to unexercised siblings (ANOVA with Tukey post-hoc, p<0.0001, n≥119, error bars = SD). (E) Cardiac failure rate in response to external electrical pacing is lower in exercise-trained, vehicle-fed MHC GS>Octβ2R RNAi RU- flies compared to age-matched, untrained siblings (Chi-squared, p = 0.0396). Vehicle-fed MHC GS>Octβ2R RNAi RU+ flies do not improve cardiac stress response after training (Chi-squared, p = 0.5367, n≥95, error bars = SEM). (F) Lysosomal activity remains similar to untrained siblings in the fat body of vehicle-fed, exercise-trained MHC GS>Octβ2R RNAi RU+ flies, but is increased in vehicle-fed, exercised RU- flies (ANOVA with Tukey post-hoc, p≤0.0304, n = 10, error bars = SEM). (G) MHC GS>Octβ3R RNAi RU- and RU+ flies fed 5μM OA or vehicle [file pgen.1008778.s008.tiff]

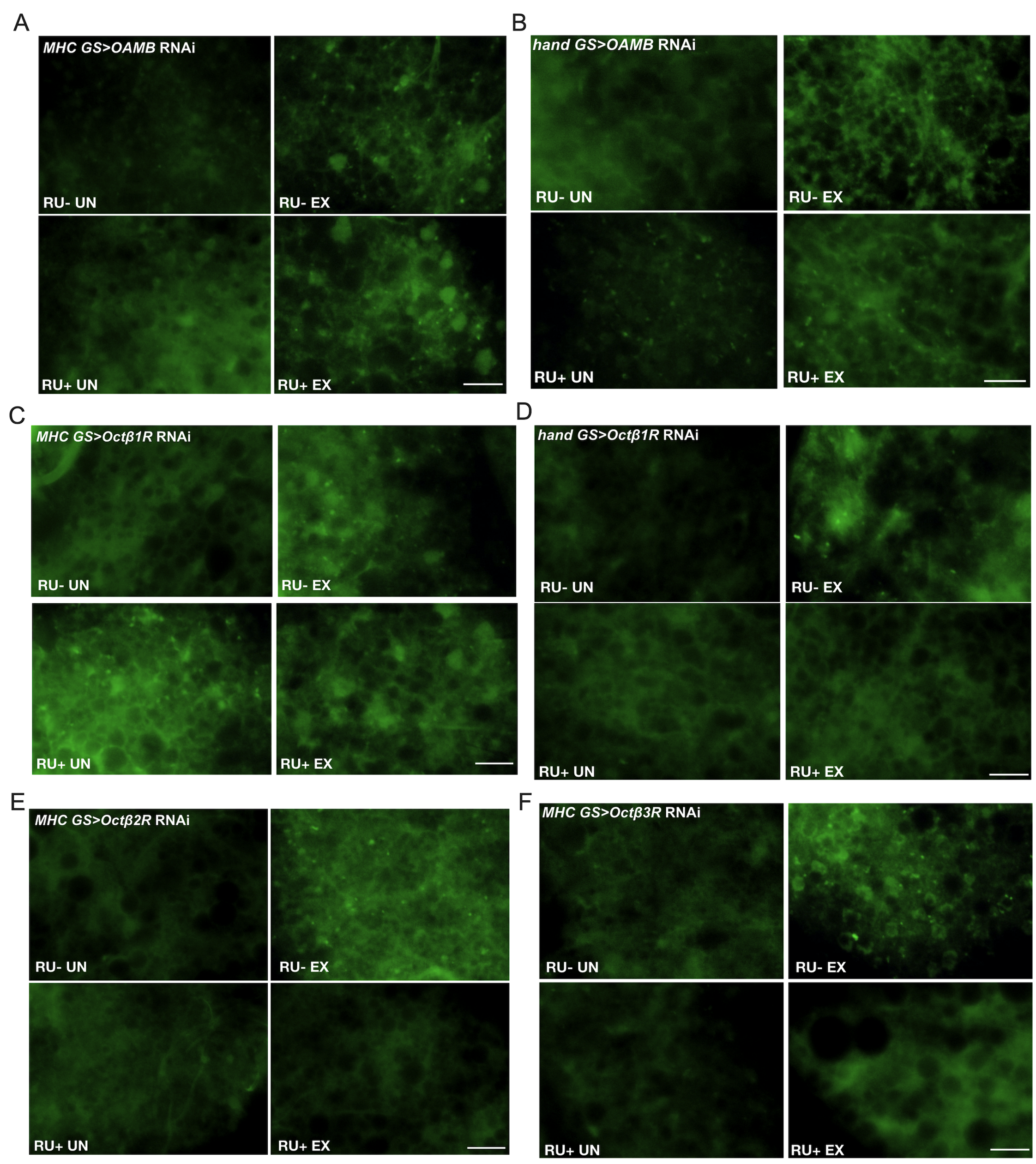

Supplement: S9 Fig — Accompanying confocal images for lysotracker quantifications in main Figs 1–3. Scale bars = 20μm. (TIFF) [file pgen.1008778.s009.tiff]
