# Supplementary material for: Alpha- and beta-adrenergic octopamine receptors in muscle and heart are required for Drosophila exercise adaptations
Source: PLoS Genet. 2020 Jun 24;16(6):e1008778. doi: 10.1371/journal.pgen.1008778 (PMC7351206; doi:10.1371/journal.pgen.1008778)
Supplement: S1 Table — (DOCX) [file pgen.1008778.s011.docx]

|  | **Runspan** | **Climbing Speed** | **Flight** **Performance** | **Pacing Resistance** | **LysoTracker** |
| --- | --- | --- | --- | --- | --- |
| ***MHC GS>Octβ2R* RNAi** | ns  ns | --  --- | ++++  + | -  -- | ----  ---- |
| ***MHC GS>Octβ3R* RNAi** | +  + | +  ++++ | ns  ns | +++  ++++ | ----  ---- |

All statistics in comparison to uninduced, unexercised, vehicle-fed background controls

**Table 1: Summary Statistics of combinatorial treatment of 5µM OA feeding plus exercise training in selected RNAi lines**

Key: Exercise Training: ++++p<0.0001

+++p≤0.001

++p≤0.01

+p≤0.05

^ns^p>0.05

^----^p<0.0001

^---^p≤0.001

^--^p ≤0.01

^-^p≤0.05

5µM OA feeding: ++++p<0.0001

+++p≤0.001

++p≤0.01

+p≤0.05

^ns^p>0.05

^----^p<0.0001

^---^p≤0.001

^--^p ≤0.01

^-^p≤0.05
