# Supplementary material for: Alpha- and beta-adrenergic octopamine receptors in muscle and heart are required for Drosophila exercise adaptations
Source: PLoS Genet. 2020 Jun 24;16(6):e1008778. doi: 10.1371/journal.pgen.1008778 (PMC7351206; doi:10.1371/journal.pgen.1008778)
Supplement: Attachment — Submitted filename: 20_02_13_Response to Reviewers.docx [file pgen.1008778.s012.docx]

**Response to Reviewers**

We thank the reviewers for their thoughtful criticisms and suggestions, which have greatly improved the paper. We have made several major changes to the data and presentation and have added a great deal more experimental controls and additional clarifying experiments. Major changes include 1) a reorganization of the figures to focus on one receptor at a time instead of focusing on one phenotype at a time, 2) all drivers used throughout the paper are now adult-induced GS drivers and all have RU- flies as negative controls, 3) we have included separate knockdowns of each receptor now and no combination knockdowns remain, 4) we now include separate qRT-PCR results for every combination of RNAi and driver both before and after chronic exercise. 5) We have added a summary figure that highlights the novel tissue-autonomous and tissue non-autonomous requirements identified in the manuscript. We believe that these changes address the reviewer’s major experimental concerns.

Reviewers also expressed more general concerns about mechanistic impact downstream of receptors. We argue that the finding that OA receptors cannot substitute for each other, even in the same tissue, and even when exogenous OA is provided, is itself a novel mechanistic insight, as it has never before been reported that specific OA receptors have individual roles to play to execute exercise adaptations in the adult fly. While we, like the reviewers, are excited about uncovering the separate downstream mechanisms associated with each receptor, this is likely to be a rather lengthy process that is too large in scope to put into this manuscript. Our primary goal is not to use exercise as a tool to study OA receptor biochemistry, rather our primary interest is uncovering mechanisms of exercise adaptation and genetic variation in exercise response. As such, the concept that levels of each OA receptor separately influence the ability to adapt to exercise is itself an impactful addition to the literature on exercise, and is entirely novel in the field of invertebrate exercise. Publication of this concept will likely encourage additional researchers to join in characterizing the downstream roles of each receptor, both in this species and in others.

We respond to each reviewer comment below, with reviewer comments in italics and response in plain font. When comments have been responded to in this opening summary, we will respond with “see opening summary”.

*Reviewer #1: In this manuscript, Sujkowski et al. build upon previous work demonstrating OA is necessary for exercise adaptations following endurance training in Drosophila by identifying which octopamine receptors are required for these adaptations. They found that OAb2R is required in adult muscle while OAMB is necessary in the heart. The authors demonstrate adrenergic signaling is key to the adaptive response to endurance exercise which is a finding that has potential to be significant and applicable in other systems and in individuals who are at risk for inactivity-related disorders. The large datasets for every paradigm is impressive and the behavioral data is rigorous and well done. The figures are constructed in a creative and easy-to-interpret manner considering the amount of data and the fairly cumbersome genotypes for each graph. In addition, the use of the Gene Switch system alleviates potential developmental issues related to reductions in OARs.*

We thank the reviewer for their encouraging comments.

*My enthusiasm is dampened by the lack of images and description of the Gal4 lines as the validity of the results weighs heavily on the tissue-restricted expression of these lines.*

We now provide references that describe each Gal4 line in detail in the methods section.

*Secondly, I realize there is an immense amount of time invested in the clear and solid behavioral experiments, however the manuscript stops short of providing a mechanism to explain how individual OARs govern exercise response. This is likely underway or will be incorporated into the next publication but it is a weakness in this manuscript.*

See opening summary

*Corrections and comments:

1. The verification of OAR transcript expression should be described earlier as these results are critical. I found myself often wondering if an individual receptor is even found in the fat body for example.*

We agree and now describe this expression in the opening section of the Results

2*. A short sentence or two describing Runspan, etc., should be provided at the start of the relevant sections so a reader does not have to look up the reference to understand the assay.*

We agree and have added such a statement beginning on line 74.

*2. A fifth receptor that is activated by OA has been described -* [*https://www.ncbi.nlm.nih.gov/pubmed/28942992*](https://www.ncbi.nlm.nih.gov/pubmed/28942992)

We now acknowledge this discovery on line 218 and include this reference.

*3. Are the Gal4 lines thoroughly characterized and do not drive expression in any other tissue? In the Materials and Methods, the hand-Gal4 and MHC-GS-Gal4 lines are listed without references and as gifts. Even if the lines are characterized, it would be useful to show the expression patterns by crossing the Gal4 lines to stinger-GFP or His2A-GFP.*

We now provide references that describe each Gal4 line in detail in the methods section. Each has been extensively used previously and carefully vetted by ourselves and others. See also more detailed discussion in reviewer #2’s section below.

*4. In the OAb2R negative geotaxis assays on page 5, it looks like the climbing phenotypes of the experimental males are not statistically different than controls. If this is correct than the sentence (line 103) should be rewritten. Currently it says the experimental flies climb faster than controls.*

This was a strange result, and our description of it was confusing. We have clarified it and the sentence now reads as follows “RNAi against *Octβ2R* in adult muscle caused unusual climbing phenotypes, with unexercised knockdown flies climbing faster than unexercised controls, but actually becoming slower when exercise-trained (Figure 3C).”

5*. At the end of page 7, a summary sentence describing the results should be provided*.

We have added the following summary sentence: “Thus, in general, phenotypes that were unaffected by a specific knockdown responded to OA feeding as normal, but adaptations that required a particular receptor were not rescued by OA feeding. This strongly suggests that different receptors are specifically required for particular exercise adaptations, and OA receptors are not interchangeable in this context.” We have also added a new figure that provides a graphical summary of the various requirements identified in the manuscript.

*6. The UAS-RNAi lines and BDSC numbers are not provided.*

We apologize for overlooking this and now provide these in the methods section.

*Typos, etc.
1. page 4, line 92. Figure instead of Figures.

2. Statistics should be added to Figure 3G and the spacing fixed.

3. There is only one * in Fig. 4D but the p-value indicates p<0.0001 as in Fig. 4A.

4. Fig. 5F has Octb1R

5. line 160, pg. 7 there are words missing in the sentence.*

These have now been corrected, although because of figure rearrangements, they no longer appear in the same places as in the first draft. The exception is the climbing speed statistics, where for reasons of spacing, statistics for relevant comparisons are contained in the legends instead. This is because the longitudinal climbing measurements encourage comparison of age effects, genotype effects, and exercise effects, as well as interactions between them. Rather than jamming all these stats into the figure, where they become confusing and crowded, we point out the stats for the specific comparisons being made in the legend.

*Reviewer #2: The manuscript by Sujkowski et al. is a follow-up of their work published in Cell Report 2017 and describes the roles of each of four known octopamine receptors in three different tissues (skeletal muscle, heart and adipocytes) in various exercise responses. There are several interesting findings such as the effects of combined OA treatment and exercise training. However, the work in the manuscript seems largely incomplete, the experiments are overall not rigorous and lack rationale (for example, there is no rationale on why the three tissues were examined), and conclusions are not supported by the presented data, offering limited significance and impact. Specific points are as follows.*

We now provide an explicit rationale for choosing these tissues for examination, based on the fact that these are the tissues where phenotypes have been observed and assessed during chronic exercise treatment.

Major points:

*1. The authors used three GAL4 lines – MHC-, hand- and S106-GAL4 for skeletal muscle, heart muscle and fat body, respectively. However, their expression patterns are not specific - for example, MHC-GAL4 is expressed in all muscle types including skeletal and cardiac muscles and Hand-GAL4 in all cardiac cells including muscle and non-muscle cells, and S106-GAL4 in the digestive system as well. Additional GAL4 lines with more restricted expression patterns would be needed to make solid conclusions on the tissue-specific roles of each receptor.*

We apologize for not addressing this more clearly in the original submission. We and others have confirmed the expression patterns of these drivers previously and published expression patterns are now referenced. MHC is expressed broadly in skeletal muscle, but is not expressed in cardiac muscle, as noted in Demontis and Perrimon 2010 and in Bai et al, 2013 and in our own observations. S106-Gal4 is expressed in the gut in larva but not adult (Kim et al, 2015), and was not induced in larvae in this study.

Hand-Gal4 is expressed in both cardial and pericardial cells and less strongly in portions of the visceral muscle. We acknowledge that some of the phenotypes seen with the driver may be partially mediated by non-muscle cardiac cells. The role of the pericardial cells in the context of exercise, if any, is not known. There is not currently in existence any driver that is both purely specific for contractile cardial cells and also expresses in all cardial cells. As knockdowns in a subset of cardial cells would have a different set of caveats, we thought it better to stick with the cardial and pericardial driver, particularly as pericardial cells are essential for wild-type heart function (e.g. Fujioka et al, 2005). It is highly unlikely that the visceral muscle expression is relevant to these phenotypes because only one OA receptor, *Octβ2R*, is expressed there (El-Kholy et al, 2015), and knockdown of that receptor with Hand-Gal4 did not block exercise. Since the only relationship to the study is a negative data point, we mention it here but did not go mention it in the text.

*2. RNAi efficacy is not documented. The authors note that each RNAi line was tested in the previous study. However, the previous study was done using da-GAL4, which is a strong driver (i.e. high expression level and ubiquitous). GAL drivers have different expression levels in different tissues, and more importantly RNAi efficacy varies depending on a tissue type. Thus, each driver on each receptor knockdown, particularly temporal knockdowns induced by 72 hrs RU486 feeding, would need to be confirmed by q-RT-PCR or in situ hybridization in the tissue type under study.*

We now include a thorough documentation of RNAi efficacy, including qRT-PCR from each specific tissue with each combination of driver and RNAi, measured separately before and after chronic exercise. These results are provided in Supplementary Material.

*3. There are genetic mutants available for each octopamine receptor. It would be of great importance to examine the genetic mutants and tissue-specific rescues to verify the noted roles of each receptor in a particular tissue.*

This is an excellent suggestion, and genetic mutants will be extremely important when dissecting separate mechanisms downstream of each receptor. Here in the context of documenting the existence of receptor specificity for the first time, we have addressed this by OA feeding single receptor knockouts, which is the inverse of the proposed experiment, and provided conclusive evidence of receptor specificity.

*4. In all result sections/figures, the following receptor knockdowns/GAL4 driver are missing.

MHC-GS-GAL4: Octb1R alone
Hand-GAL4: Octb2R, Octb3R alone
S106: Octb1R, Octb2R and OAMB*

We agree that these were important experiments, and they have now been added.

*4.1. When Octb3R/1R knockdown causes a phenotype, the conclusion cannot be made as to whether Octb3R or Octb1R alone, or combined actions of Octb1r and Octb3r are important in the absence of individual Octb1R and Octb3R knockdowns.*

We agree and have removed this double knockdown from the revised manuscript, replacing it with the ones suggested by the reviewer in the previous comment.

*4.2. There is no rationale for knocking down Octb1R and Octb3R together. If the goal is to examine the effects of multiple receptor knockdowns, other receptor combinations are missing.*

See above

*4. 3. For MHC and S106, GS-GAL4 is used for adult stage-specific knockdown. While Hand-GS-GAL4 is available, Hand-GAL4 is used in the study and no rationale is provided for it.*

We agree and have removed hand-gal4 and replaced it with GS-hand-gal4.

*5. In the study on adaptation to chronic exercise, the duration of RU486 treatment is not noted. Regardless whether RU486 was treated for 3 days or throughout chronic exercise training (25 days), the endurance test on “acute” exercise should be done on Day 25 with the same RU486 treatment regime since the levels of octopamine receptor knockdowns are likely different on Day 5 (acute) and Day 25 (chronic). Otherwise the role of the receptors on acute and chronic exercise cannot be compared.*

RU486 treatment is continuous throughout adulthood and is intended to test the requirement for each receptor during the exercise training period. Refraining from inducing the RNAi until the last 3 days would not accomplish this goal, as the animals would have been experiencing 18 days of exercise training already before RNAi induction, which is already sufficient to produce some adaptation (Piazza et al, 2009).

*6. The experiments on the combined OA treatment and chronic exercise training are interesting. However, there is no rationale (or underlying hypothesis) for the study, the data are difficult to see and understand, and the interpretation and discussion are superficial.*

We have added much more text in this section of results to detail the rationale and added a summary at the end of the section to better explain the results.

*7. The qRT-PCR study is not well described. For example, it is unclear whether the Ex analysis was done after acute or chronic exercise, which is important but not noted. Also the analyses were noted to be done on duplicate samples but 6 sample symbols shown in the graphs (duplicate biological replica and triplicate on each replica?). There are large variations in the data and it seems that additional sample size would be helpful to support the conclusion. Also, complete qRT-PCR analyses on all knockdowns in all tissue types used for behavioral experiments (only subsets were done) would be needed to make solid conclusions.*

All qRT-PCR combinations are now included and presented in a new figure format in the supplemental material.

*8. Discussion on the data obtained using global (TubGS RNAi in the previous paper) versus tissue-specific (this paper) knockdowns of individual octopamine receptors would be helpful.*

We have included additional text discussing this, starting on line 249. In general, the TubGS RNAi for each receptor represents the additive phenotype of the various tissue-specific RNAis, further supporting the consistency of the results.

*Minor points:

1. Materials and methods

1.1. The authors note that the behaviors are scored blindly when possible. It is important to note which behaviors are scored blindly and which behaviors are unable to score blindly.

1.2. Information on most fly strains used in the study is missing

1.3. Inconsistent formatting in materials and method for product info (i.e. the city and state information is noted in some but not all)

2. Writing/figures/tables

2.1. Non-italicized Drosophila (P2 L39); Fig S1C, S2A, S2C - genotypes are not italicized

2.2. mislabeling in Fig 2E: it appears hand>OctB1R for both red solid line and red dashed line – missing UN and EX

2.3. In P4 L89-91, it states “Neither heart specific Octβ1R RNAi nor adult fat-body specific RNAi against Octβ3/β1R reduced exercise dependent improvements in endurance (Figures 2E, H).” but Fig 2H depicts B3 knockdown only.

2.4. “Crowded” labeling in figures, especially genotype labels (see fig 4E, 4F, 5E, 5F, etc.)

2.5. In P7 L89-91, it states “…knockdown of OAMB, OctB3R or OctB1R in muscle…” but Fig 6C only depicts OAMB knockdown.

2.6. Statistical analyses are missing in several places (e.g. figure 3)

2.7. Table 1 - mixed symbols (asterisk and minus sign) and letters make the content difficult to understand.

2.8. Table 2 – incomplete and not so informative*

The extensively revamped manuscript has all-new figures and a newly organized text. We believe the accumulation of all these changes addresses the issues pointed out by the reviewer, and hope that the new version is more clear.

*Reviewer #3: A previous work by Sujkowski et al. showed that increased activity of octopaminergic neurons is both necessary and sufficient for exercise adaptations in Drosophila. This manuscript is a follow up on that work and here the authors examined the requirements for each of the 4 octopamine receptors in different tissues on the exercise response. They found that each of the octopamine receptors is required for parts of the exercise response in a tissue-specific way and specific to particular phenotypic outputs within the broad suite of exercise responses. They also identify that octopamine signaling in one tissue can affect other tissue phenotypes. While the findings showing the detailed dissection of the input of each octopamine receptor on exercise response is appreciated, the primary concern is that this work is largely descriptive and has not carried things far enough forward, in terms of scope/mechanism and novelty, that would elicit strong general interest from the general readership of the journal here. The study at this stage may be more appropriate for a more specialized journal.*

We appreciate the concern and have argued in the opening summary why we feel the advance here is substantially novel.

*Concerns:
1) Figure 1 – The extent of RNAi knockdown of the 4 octopamine receptors using MHC GS, Hand, and S106 should be determined by QRT-PCR or muscle/heart immunostaining. Even though these RNAi efficiencies have been previously confirmed, as stated.*

See response to the similar comment from Reviewer #2 above.

*2) Figure 6 – The impact on autophagy/lysosomal activity by the various tissue knockdowns of the octopamine receptors has to be studied in more detail using more indicators/markers of autophagy/lysosome, not just Lysotracker.*

We have added Atg8a to Ag8b ratio in exercised and OA-fed flies, along with fat specific knockdowns to Figure 4.

*3) All the inhibitory manipulations were performed using genetic knockdowns, not knockouts. Therefore the genetic backgrounds are not “clean” (i.e. there are residual proteins) which could confound the interpretations. For eg., when knocking down one octopamine receptor in one tissue does not affect runspan while knocking down the same/another receptor in another tissue does, is it really because one is dispensable while the other is not, or because one is more depleted than the other?*

This is a valid concern with the previous version. Now that we have provided separate tissue-specific qRT-PCR data for each receptor in each knockdown, and provided exogenous OA to analyze whether remaining OA receptors can substitute for the knockdown of a single receptor, we believe these experiments address this issue much better in the revised version.

*4) Some mechanistic insights on the tissue non-autonomous effects of cardiac OAMB knockdown would heighten the enthusiasm for the work, especially given that OAMB reduction in the heart produces profound cell-autonomous effects of receptor activation during endurance exercise.*

We are excited to continue forward with the suggested experiments, but given the large amount of data in this manuscript and the laborious nature of these behavioral experiments, we feel that these experiments should come in a separate manuscript, as it will require an extensive set of experiments to complete that story. We also intend to follow up by examining downstream effects of each separate receptor, each with its own story, but each will require extensive experimental work that is beyond the scope of this paper, which already contains a substantial body of results.
