# Supplementary material for: Alpha- and beta-adrenergic octopamine receptors in muscle and heart are required for Drosophila exercise adaptations
Source: PLoS Genet. 2020 Jun 24;16(6):e1008778. doi: 10.1371/journal.pgen.1008778 (PMC7351206; doi:10.1371/journal.pgen.1008778)
Supplement: Attachment — Submitted filename: Reviewer 3.docx [file pgen.1008778.s013.docx]

Overall, the authors have made improvements to the manuscript in terms of experimental and editorial additions, although the mechanistic aspects are still lacking. In light of the absence of mechanisms, more weight would have to be given to the phenotypic analyses. I would recommend two further minor points to increase the rigor of the analyses.

1) While the authors have measured the RNAi knockdown efficiencies for each gene driven by different Gal4 drivers (Supplementary Figure 1), there is no mention of the extent/degree of knockdown in the text. These knockdown efficiencies should be explicitly described in the text upon the first mention of the gene knockdown (for eg., Using X-Gal4 to induce knockdown of OAMB, we detected a Y% reduction in OAMB expression in adult muscles ….).

2) The authors showed only the quantification of lysotracker. Representative confocal images should be included in the figures with the quantification residing next to the images for better visual understanding.
