# Supplementary material for: Alpha- and beta-adrenergic octopamine receptors in muscle and heart are required for Drosophila exercise adaptations
Source: PLoS Genet. 2020 Jun 24;16(6):e1008778. doi: 10.1371/journal.pgen.1008778 (PMC7351206; doi:10.1371/journal.pgen.1008778)
Supplement: Attachment — Submitted filename: Response To Reviewers 3-20.docx [file pgen.1008778.s014.docx]

**Response To Reviewers**

We thank the reviewers for their thoughtful comments, criticisms and support. Below, we respond to each critique individually. Reviewer comments are in Arial and our responses are in Calibri.

Reviewer #1: Minor note, in the response to reviewers it is really helpful to provide line numbers of the corrections and new figure numbers instead of “new figure” to help the reviewer quickly identify the change and the text context of the change.
However, congratulations on this exciting paper.

We thank the reviewer for their encouraging comments. In this response, we have included the line numbers of each change and apologize for not doing so in the previous one.

Reviewer #3

1.While the authors have measured the RNAi knockdown efficiencies for each gene driven by different Gal4 drivers (Supplementary Figure 1), there is no mention of the extent/degree of knockdown in the text. These knockdown efficiencies should be explicitly described in the text upon the first mention of the gene knockdown (for eg., Using X-Gal4 to induce knockdown of OAMB, we detected a Y% reduction in OAMB expression in adult muscles ….).

This is a great suggestion, and we now describe the knockdowns in the text. Because all the knockdowns are in one supplemental figure, we now describe them as a group at the very beginning of the results section where we describe the general strategy, and we refer to the figure for specific details of each one. The description appears on lines 93-98.

2) The authors showed only the quantification of lysotracker. Representative confocal images should be included in the figures with the quantification residing next to the images for better visual understanding

We now include a sample image of a confocal projections for each quantitated group. The most relevant ones have been placed directly in the main figures, in panels 4G, 5F and 6F. The rest have been gathered into a new supplemental figure, Supplemental Figure S9.

Reviewer #4

The authors have addressed almost all of the major points brought up by the three reviewers, however, there is one comment that in my opinion was insufficiently addressed. On reviewer 2 comment 8, the authors should elaborate further on the discrepancies between the phenotypes observed using global KD (Cell reports 2017) versus tissue specific KD (this revised manuscript) of individual OA receptors. For example, address questions including:
A. Why ubiquitous Octb3R KD versus fat body Octb3R KD yielded different results in the lysosomal activity? This is especially important since it is one of the major findings attributed to Octb3R function in exercise adaptation.
B. Why ubiquitous Octb1R KD versus either muscle or fat body Octb1R KD have different effects on endurance, as measured by runspan? The authors only address flight.
C. Why ubiquitous Octb2R KD affects all exercise adaptations (Cell reports 2017) yet in this was not the case for tissue specific Octb2R KD? Especially address the discrepancy on flight.
This section in the discussion can touch on the the known signaling pathways that the different in phenotype expression and begin to describe possible molecular mechanisms downstream of OA receptors affected by exercise. This, in part, may address the similar concerns expressed by the other reviewers.

We agree that these are important and fascinating questions that we plan to work on in more detail in upcoming years. We cannot answer these questions with certainty with the data that we have in this manuscript, but we have added an additional paragraph of discussion postulating some possibilities that could explain these differences. In the context of this discussion, we also mention downstream pathways that may be differentially repressed depending on the extent of knockdown, as we agree with the reviewer that the answer to these questions is likely to be found in this area. The new discussion appears in lines 285-304.

The experiment using with OA feeding in the flies with OA receptor KD (Octb2R and Octb3R) in muscle are great. These results indeed facilitated the identification of receptor requirement or functional substitution for exercise responses. The data shown is on the Octb2R and Octb3R in muscle tissue, a great proof of concept. However, in lines 228-231, this statement in my opinion, is too bold. This generalized statement can be easily misinterpreted to encompass all OA receptors in different tissue types (e.g. OAMB and Octb1R). Unless, there are OA feeding data on all individual OA receptor KDs, the authors should amend this statement.

This is a good point, and we have revised the statement to say “not all OA receptors are interchangeable” and changed the word “different receptors” to “some receptors”, since we agree with the reviewer that the previous wording was overly inclusive. The new statements can be found on lines 222-225.

In line with the previous comment, the authors argue the novel mechanism that “…OA receptors cannot substitute for each other, even in the same tissue…” is indeed true for exercise adaptations. This concept, however, has been demonstrated in the female reproductive system epithelium in Lim et. al., 2014 (ref 41), where the OAMB could not functionally substitute for Octb2R in the to restore fecundity yet OAMB can partially substitute for Octb2R to restore the ovulation phenotype of the Octb2R mutant females. Both OAMB and Octb2R are expressed in the female oviduct epithelium and are important for female reproduction. The authors may reference this to support their findings and hypothesis on the similar phenomenon observed for exercise adaptations.

We agree and we have mentioned this along with the reference on lines 246-247.

Are the specific OA neurons required for exercise adaptations known or mapped? If so, does it involve all or subset (e.g. Tdc2+, VUM, VPM or APL)? Do these OA neurons send axonal projections to the different tissue types (muscle, heart or fat body) tested? Alternatively, is OA released into the fly circulation? The authors can add this information in the discussion.

We made an effort to identify a subset of OA-ergic neurons that were responsible for the exercise response as part of the transformer series of experiments in our 2017 paper, and those experiments were summarized in a supplemental table in that paper. No subset of OA-ergic neurons out of 5 drivers from the Janelia Farms neural project that we tested replicated any of the effects of the broad Tdc2 –Gal4. Since these drivers were in very different subsets of OA-ergic neurons, we concluded that this phenotype was likely not dependent on a particular circuit, and instead required release of OA into the circulation. This is consistent with the findings in the current manuscript that particular receptors in various tissues have roles to play, and is also consistent with the role of norepinephrine in human exercise. We thank the reviewer for bringing out this point that we neglected to mention in the discussion, and we have now added a short discussion about this in lines 260-264.

In lines 186-188, the statement “…trends toward lower levels…”, in my opinion, is misleading. There is no statistics information presented to suggest the “lowering” trend. The authors should rephrase this sentence.

We have rewritten this sentence for better clarity, added more information about the labeling in the legend for figure 4, and we also added stats into the quantification in figure 4I. The new language in the text is in lines 180-182.

Lines 197-198 state, “summary of OA-feeding+exercise results is in Table 1”, there is no Table 1 either the manuscript doc, pdf, or supplemental doc. This should be clarified.

We apologize for this error, we neglected to add the Table to the upload in the previous submission, it is there now.

Lines 268-269 state, “OAMB, the only Drosophila α-adrenergic OA-receptor…” is incorrect. There is another one, the Octα2R that the authors reference – Qi et. al. 2017 (ref 48). This sentence needs to be revised.
We agree and have removed the clause “the only…receptor” from line 268.

In line 681, missing close parenthesis after “(K”.
We added the close parenthesis, it is now on line 708

The statistics information for the cardiac pacing experiment is missing in the methods section.

We have added the stats info to the methods on lines 398-400

The information for S106 GS is missing from the fly stocks section

This has been added on line 322.

The labeling in Figure 4G is not easy to follow especially the “WT(-)(+)” on top of the blot image – an explanation in the figure legend would be helpful.

We added more info to the figure legend for the former figure 4G, now figure 4H to clarify on lines 477-481, and also added stats directly into figure 4I. The + and – refer to RU treatment, while ex and un are separated by red and black.

The Figure 7 legend states, “…Cell non-autonomous effects…indicated by colored arrows.”, but there three sets of colored arrows in the summary: (1) denoting increased OA, (2) arrows denoting OA binding to different OA receptors and (3) cell non-autonomous effects. This should be clarified.

We added clarifying language to the figure 7 legend about the arrows in lines 626-629.

The first two sentences of the abstract are identical to the authors’ previous publication on Cell reports 2017. The authors should rephrase/reword these sentences

We changed the sentences to read “Endurance exercise has broadly protective effects across organisms, increasing metabolic fitness and reducing incidence of several age-related diseases. *Drosophila* has emerged as a useful model for studying changes induced by chronic endurance exercise, as exercising flies experience improvements to various aspects of fitness at the cellular, organ and organismal level.”
